# Contained Juxtarenal Aortic Perforation in the Setting of Severe Warfarin-Associated Coagulopathy

**DOI:** 10.1016/j.jaccas.2026.108398

**Published:** 2026-05-19

**Authors:** Besher Shami, Riyadh Saif, Hsu Yadana, Iman Mousselli, Imad Samman Tahhan, Pradeep Manoharan

**Affiliations:** a Department of Medicine, Berkshire Medical Center, Pittsfield, Massachusetts, USA; b Department of Internal Medicine, Wellington Regional Medical Center, Wellington, Florida, USA; c Department of Cardiology, Berkshire Medical Center, Pittsfield, Massachusetts, USA

**Keywords:** anticoagulation, aorta, hypertension

## Abstract

**Introduction:**

Severe warfarin-associated coagulopathy is a known cause of major bleeding; however, its relationship with large-vessel structural complications remains poorly defined. Spontaneous rupture or perforation of a nonaneurysmal abdominal aorta is rare and may present atypically, particularly when hemorrhage is contained.

**Case Presentation:**

A 90-year-old woman on chronic warfarin therapy presented with abdominal pain and an unmeasurable international normalized ratio. Computed tomography demonstrated a contained juxtarenal aortic perforation with a retroperitoneal hematoma, whereas prior imaging weeks earlier showed no aneurysm. Anticoagulation was reversed and hemodynamic control was initiated. Because of high operative risk, she was transitioned to comfort-focused care.

**Discussion:**

This case highlights acute aortic perforation in the absence of prior aneurysmal disease. Although anticoagulation is unlikely to directly cause rupture, it may exacerbate hemorrhage following structural disruption, likely related to underlying atherosclerotic or ulcerative pathology.

**Conclusion:**

Early imaging, prompt reversal, and hemodynamic control are essential, although outcomes remain poor without definitive intervention.

## Visual Summary

**Figure:**
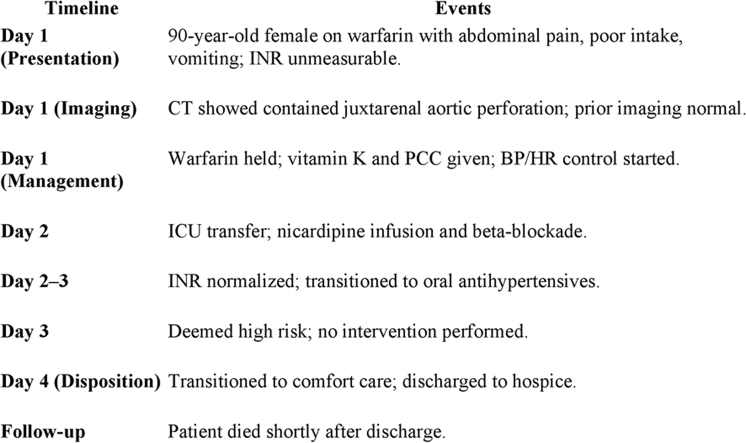

**Visual Summary:**
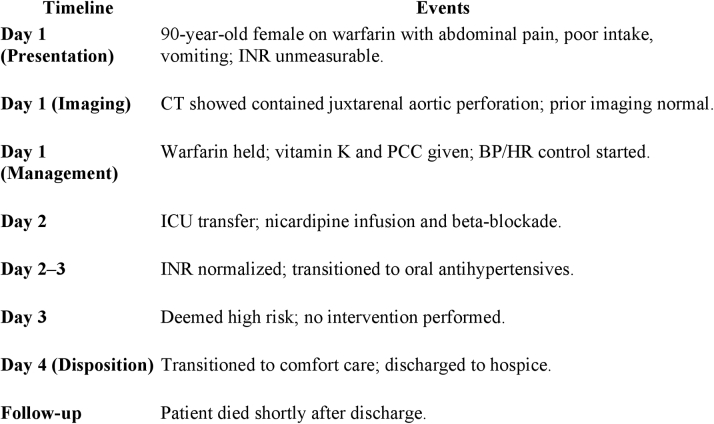
Clinical Timeline of Contained Juxtarenal Aortic Perforation in the Setting of Severe Warfarin-Associated Coagulopathy, Highlighting Presentation, Diagnosis, Reversal of Anticoagulation, Hemodynamic Management, and Transition to Comfort Care BP = blood pressure; CT = computed tomography; HR = heart rate; ICU = intensive care unit; INR = international normalized ratio; PCC = prothrombin complex concentrate.

Severe warfarin-associated coagulopathy is a common clinical challenge; however, its association with large-vessel structural complications remains poorly characterized. Warfarin remains widely used for long-term anticoagulation despite its narrow therapeutic index and susceptibility to multiple pharmacologic and physiologic interactions.^1^ Supratherapeutic anticoagulation is a well-recognized cause of major bleeding, most commonly involving the gastrointestinal tract, intracranial space, and genitourinary system. The risk of bleeding increases significantly with elevated international normalized ratio (INR) levels.

In older patients, these effects are amplified, resulting in greater variability in anticoagulation control and increased susceptibility to overanticoagulation. Multiple factors influence warfarin metabolism, including dietary vitamin K intake, drug interactions, genetic variability, and alterations in gut microbiota. Antibiotic exposure further contributes by reducing intestinal vitamin K production and potentiating warfarin activity.^2^

Acute aortic syndromes are typically associated with aneurysmal disease, hypertension, or structural aortic pathology.^3^ However, spontaneous rupture or perforation of a nonaneurysmal aorta is rare and increasingly recognized, particularly in the setting of focal atherosclerotic disease or penetrating ulceration.^4^, ^5^, ^6^ When rupture is contained within the retroperitoneum, patients may present subacutely with relatively preserved hemodynamics, further complicating diagnosis.^5^

The relationship between anticoagulation and acute aortic pathology remains incompletely understood. Although anticoagulation alone is unlikely to cause aortic rupture, it may significantly amplify hemorrhage once structural disruption occurs.

We present a case of contained juxtarenal aortic perforation in the setting of extreme warfarin-associated coagulopathy, with prior imaging demonstrating no aneurysmal disease.

## Case Presentation

A 90-year-old woman with history of recurrent pulmonary embolism on chronic warfarin therapy since 2019, prior cerebrovascular accident, hypertension, hypothyroidism, polymyalgia rheumatica on chronic prednisone, inferior vena cava filter placement, and rectosigmoid adenocarcinoma status post right hemicolectomy, presented to the emergency department with progressively worsening abdominal pain of 1-week duration.

The pain was initially localized to the mid-abdomen and subsequently radiated to the lower chest and back at a similar level. She also reported poor oral intake, generalized weakness, intermittent lightheadedness, and persistent vomiting for 5 days before presentation. She denied fever, cough, shortness of breath, lower extremity pain, numbness, recent trauma, surgery, or major cardiovascular events.

Her home medications included warfarin (2.5 mg daily), furosemide, labetalol, levothyroxine, losartan, prednisone (5 mg daily), and multivitamins. She reported adherence to her warfarin regimen with regular INR monitoring through her primary care physician. Her most recent INR 1 week before admission was 1.4 (prothrombin time [PT] 14 seconds), and approximately 3 weeks prior she had completed a course of antibiotics at a nursing facility. She denied recent dietary changes or medication adjustments.

On initial evaluation, the patient appeared frail but was alert, oriented, and in no acute distress. Vital signs were notable for blood pressure of 158/69 mm Hg, heart rate 74 beats/min, respiratory rate 14 breaths per minute, and she was afebrile. Cardiovascular examination revealed a regular rhythm without murmurs. Lungs were clear to auscultation. Abdominal examination demonstrated tenderness in the periumbilical and epigastric regions without rebound or guarding.

Laboratory evaluation revealed potassium 3.2 mmol/L, bicarbonate 14 mmol/L, lactate 2 mmol/L, platelet count 242 × 109/L, PT >90 seconds, and an unmeasurable INR. Liver function tests were within normal limits (aspartate aminotransferase 10 U/L, alanine aminotransferase <7 U/L), and fibrinogen was elevated at 617 mg/dL. Initial hemoglobin was 11 g/dL, which subsequently trended downward during hospitalization. Laboratory findings are summarized in Table 1.

**Table 1:** Presentation vs Discharge Labs

Laboratory Testing	At Presentation	Repeated	After Treatment	At Discharge	Reference Range
Hemoglobin	11	10.8	9.7	8.4	12-16 g/dL
Platelets	242	236	198	186	140-400 K/μL
Prothrombin time	>90	>90	12.8	13	9.5-12.1 seconds
International normalized ratio	Unmeasured	Unmeasured	1.2	1.3	
Partial thromboplastin time	68	67			22-32 seconds
Alanine aminotransferase	<7			8	<34 IU/L
Aspartate aminotransferase	10			12	11-34 IU/L

Computed tomography (CT) of the abdomen and pelvis demonstrated interval development of abnormal appearance of the proximal abdominal aorta immediately below the origin of the superior mesenteric artery. There was discontinuity of mural wall calcifications with extension of contrast beyond the expected aortic wall and surrounding retroperitoneal hematoma, findings concerning for contained juxtarenal aortic perforation (Figure 1).

**Figure 1:**
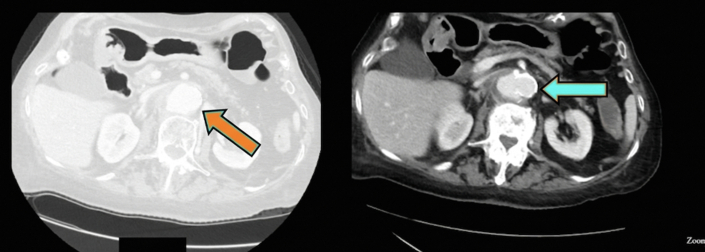
Computed Tomography of the Abdomen and Pelvis Demonstrates Findings Suggestive of Retroperitoneal Hematoma and Contained Juxtarenal Aortic Perforation The orange arrow (left image) points to the contained contrast-filled outpouching/perforation arising from the juxtarenal abdominal aorta, while the blue arrow (right image) highlights the periaortic hematoma/contained leak adjacent to the aortic wall.

Prior CT imaging performed 1 month earlier demonstrated atherosclerotic disease without aneurysm (Figure 2) and CT angiography performed approximately 5 weeks prior similarly showed no evidence of aneurysm or dissection (Figure 3), confirming that this represented an acute vascular event in a previously nonaneurysmal aorta.

**Figure 2:**
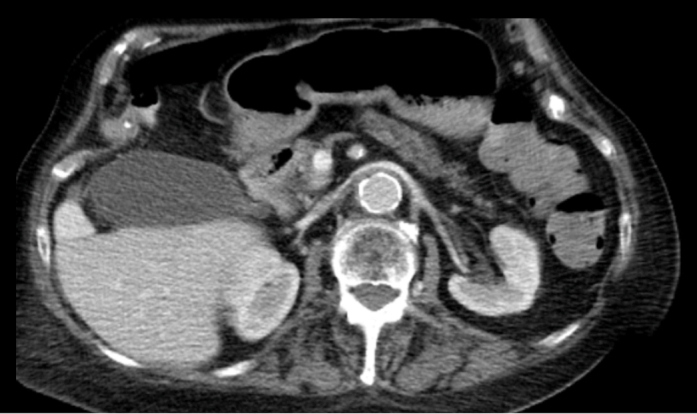
A Prior Computed Tomography Scan With No Aneurysm or Hematoma

**Figure 3:**
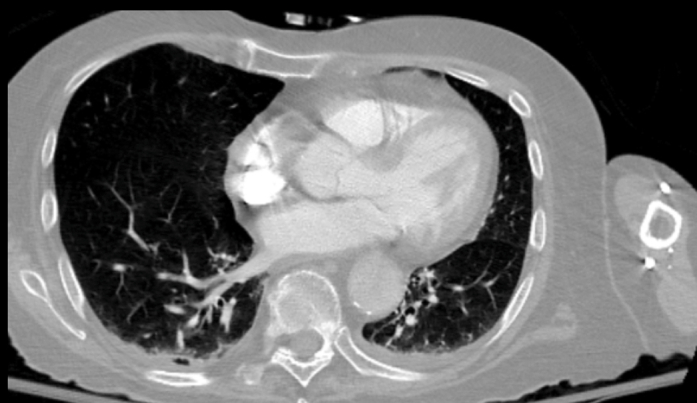
A Prior Computed Tomography Angiogram With No Evidence of Aneurysm or Dissection

## Differential Diagnosis and Diagnostic Rationale

In evaluating the etiology of this catastrophic event, we considered both the systemic hematological derangement and the localized structural failure of the aorta.

### Etiology of coagulopathy

The abrupt elevation in INR was likely multifactorial. Although hepatic dysfunction was excluded by normal transaminases and disseminated intravascular coagulation was deemed unlikely due to preserved platelet counts and fibrinogen levels, the unmeasurable INR was markedly disproportionate to the patient's clinical status. The most probable contributors included a synergistic effect of advanced age, recent antibiotic exposure, and reduced oral intake due to gastrointestinal distress, which cumulatively overwhelmed the patient's homeostatic vitamin K–dependent pathways.

### Etiology of the aortic lesion

In the absence of pathologic or intravascular imaging confirmation, several alternative etiologies for the acute focal aortic lesion were considered:

• Malignancy-associated injury: Given the history of colorectal adenocarcinoma, tumor invasion or an aortoenteric fistula were considered. However, the juxtarenal location (L1-L2) was anatomically distant from the prior right hemicolectomy site. Furthermore, CT imaging lacked features suggestive of aortoenteric fistula, such as ectopic gas, disruption of the aortoenteric fat plane, or associated inflammatory changes, and demonstrated no mass-like lesion or lymphadenopathy to suggest tumor invasion.
• Radiation and infection: Radiation-induced vasculopathy was unlikely given the absence of prior abdominal radiation. Similarly, an infectious (mycotic) etiology was considered less likely because of the absence of fever, leukocytosis, or a suggestive clinical prodrome. In addition, CT imaging did not demonstrate features typically associated with mycotic aneurysm, such as periaortic inflammation, soft tissue stranding, or irregular saccular dilation. The presence of dense mural calcification at the site of disruption further supports a chronic atherosclerotic substrate rather than acute infectious destruction.
• Primary aortic neoplasm: Primary aortic tumors are exceedingly rare and typically present as mass-like lesions with intraluminal or extraluminal extension. In this case, there was no evidence of a mass on current or prior imaging performed 5 weeks earlier, making this diagnosis unlikely.

### Diagnostic rationale for penetrating atherosclerotic ulcer

In the absence of postmortem data, a penetrating atherosclerotic ulcer remains the most plausible etiology. This is supported by CT findings demonstrating focal discontinuity of mural calcifications with contrast extravasation beyond the expected aortic contour, consistent with localized plaque disruption. Importantly, there was no evidence of an intimal flap to suggest dissection, nor mass-like features to suggest neoplasm. The rapid transition from a stable, nonaneurysmal aorta on imaging 5 weeks before a contained perforation further supports an acute mechanical failure of a preexisting atherosclerotic plaque. Although these findings are most consistent with penetrating atherosclerotic ulcer, they are not pathognomonic, and definitive differentiation from other etiologies cannot be established without histopathologic or intravascular imaging correlation.

## Management

The patient was initially admitted to the progressive care unit, where early blood pressure control was initiated with intravenous labetalol (5 mg) and 2 doses of intravenous enalaprilat (1.25 mg). Despite these interventions, systolic blood pressure remained above the desired target range (80-120 mm Hg), prompting escalation of care and transfer to the intensive care unit (ICU) for continuous hemodynamic management.

In the ICU, a continuous intravenous nicardipine infusion was initiated at 10 mg/h and titrated to 5 mg/h as blood pressure improved. Labetalol was administered as needed for additional blood pressure control, and intermittent metoprolol (Lopressor) was used for heart rate control, targeting a heart rate below 60 to 70 beats/min.

Over the subsequent days, the patient was gradually transitioned to oral antihypertensive therapy. Labetalol was initiated at 50 mg twice daily and later increased to 100 mg twice daily, which was continued for the remainder of hospitalization. Intravenous antihypertensive therapy was discontinued once adequate blood pressure control was achieved. Losartan 25 mg daily was also introduced during hospitalization with good effect. Figure 4 shows a summary of blood pressure trends and antihypertensive treatment timeline.

**Figure 4:**
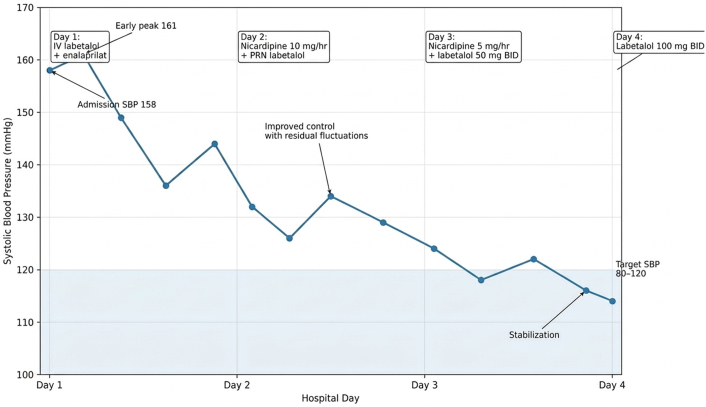
Blood Pressure Trend and Antihypertensive Treatment Timeline BID = twice daily; IV = intravenous; PRN = as needed; SBP = systolic blood pressure.

Anticoagulation was immediately held on admission. Repeated INR and PT showed the same values of presentation values. Given the severity of coagulopathy, the patient received intravenous vitamin K (phytonadione 10 mg) and 4-factor prothrombin complex concentrate (1,130 U). Clinical improvement in coagulation status was observed following reversal therapy, with INR and PT reaching 1.3 and 13 seconds, respectively. Figure 5 summarizes INR and PT levels through hospitalization.

**Figure 5:**
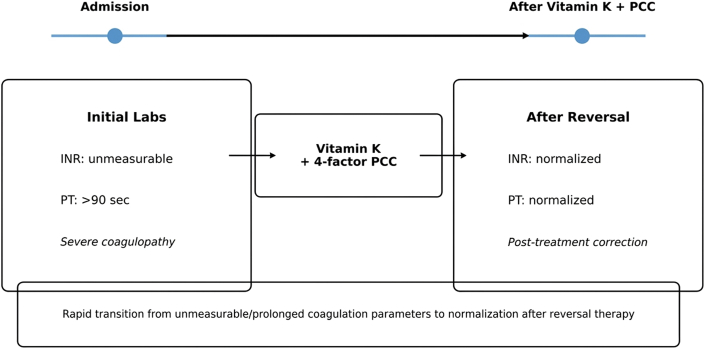
Correction of Coagulopathy After Reversal Therapy INR = international normalized ratio; PCC = prothrombin complex concentrate; PT = prothrombin time.

Strict blood pressure and heart rate control were prioritized throughout hospitalization to reduce shear stress on the aortic wall and limit progression of the contained perforation.

Vascular surgery consultation confirmed that the lesion was located at the level of the renal arteries and superior mesenteric artery, consistent with a complex juxtarenal aortic perforation. The decision to forgo definitive intervention was based on both anatomic and patient-specific considerations. The juxtarenal location precluded standard endovascular aortic repair because of the absence of an adequate proximal landing zone. Endovascular exclusion would have required a customized fenestrated or branched endograft to maintain visceral perfusion; however, these devices are not readily available in emergent settings and require specialized planning and institutional expertise not accessible at the presenting center.

Open surgical repair would have required suprarenal or supraceliac aortic clamping, which is associated with substantial perioperative risk, particularly in older patients with significant comorbidities. In this case, advanced age, frailty, and multiple comorbid conditions—including prior malignancy and chronic anticoagulation—further increased operative risk.

Although transfer to a tertiary care center for advanced endovascular intervention was considered, the patient's overall clinical condition and goals of care limited the feasibility of this approach. Following multidisciplinary discussion, nonoperative management with strict hemodynamic control and reversal of anticoagulation was determined to be the most appropriate course.

Pain control was achieved using as-needed morphine and tramadol. The patient remained hemodynamically stable throughout her ICU course and was subsequently transferred to the medical floor. Arrangements were made for hospice care, and the patient was discharged to a skilled nursing facility with hospice services. Unfortunately, she died shortly thereafter.

## Discussion

Severe warfarin-associated coagulopathy may occur in the setting of advanced age, reduced oral intake, and recent antibiotic exposure, all of which can potentiate anticoagulant effect through depletion of vitamin K stores and altered metabolism.^2^ The patient's markedly elevated PT with unmeasurable INR reflects an extreme but plausible manifestation of compounded risk factors.

Warfarin-associated bleeding most commonly involves gastrointestinal or intracranial sites, with retroperitoneal hemorrhage less frequently reported, although the source of bleeding in these reports is venous or arises from small arterial branches. However, involvement of major arterial structures such as the aorta is exceedingly uncommon.^6^^,^^7^

In contrast, spontaneous rupture of a nonaneurysmal abdominal aorta is a rare but increasingly recognized entity. The pathogenesis is thought to involve focal structural weakness of the aortic wall, most commonly related to advanced atherosclerosis or penetrating atherosclerotic ulceration. Penetrating ulcers result from disruption of atherosclerotic plaques with extension into the media, which may progress to intramural hematoma, pseudoaneurysm formation, or frank rupture.^4^^,^^5^ Importantly, these lesions may occur in aortas that are not dilated, highlighting that aneurysmal size alone is not the sole determinant of rupture risk.

Several case reports have described spontaneous rupture of nonaneurysmal abdominal aortas, highlighting the heterogeneity of presentation and underlying mechanisms. Goldstein et al^4^ described rupture of a nonaneurysmal infrarenal aorta in the setting of advanced atherosclerotic disease, emphasizing that focal wall weakening may occur even in the absence of aneurysmal dilation. Our case similarly demonstrates focal weakness in a nondilated vessel but is distinguished by the extreme coagulopathic state, which likely accelerated the resulting hemorrhage. Kainuma et al^5^ reported successful endovascular repair of a spontaneous rupture in a nonaneurysmal infrarenal aorta, demonstrating that acute rupture can occur without preceding radiographic abnormalities. Although our patient also had an acute rupture, the juxtarenal location at the level of the renal and superior mesenteric arteries presented a far higher technical barrier for repair compared with the infrarenal location described by Kainuma et al.^5^ Thalheimer et al^6^ described spontaneous perforation of a nonaneurysmal aorta due to a penetrating atherosclerotic ulcer, supporting the role of localized plaque disruption in the pathogenesis of rupture. Similarly, Vasquez et al^7^ reported spontaneous rupture secondary to a penetrating atheromatous ulcer, further reinforcing that focal ulcerative disease may predispose to catastrophic failure in otherwise nondilated aortas. Our imaging findings of mural calcification discontinuity are compatible with focal atherosclerotic or ulcerative wall pathology. Although the patient's profound coagulopathy may have worsened the extent of hemorrhage, it cannot be concluded that it initiated the aortic wall disruption. In contrast, Nasr and Elhassan^3^ reported warfarin-associated spontaneous retroperitoneal hemorrhage originating from the renal vein, illustrating that although severe coagulopathy can lead to significant retroperitoneal bleeding, involvement of major arterial structures such as the aorta is exceedingly uncommon. Our case is particularly rare because it involves direct aortic perforation rather than a venous source. However, this observation should not be interpreted as evidence that warfarin toxicity directly caused large-vessel structural failure; rather, severe coagulopathy likely complicated the hemorrhagic presentation of an underlying aortic lesion. Collectively, these reports demonstrate that rupture can occur in nonaneurysmal aortas and may present acutely with abdominal pain or hemodynamic instability, supporting the concept that localized wall vulnerability—rather than global dilation—may predispose to catastrophic failure. In the present case, prior imaging demonstrated no aneurysm or focal abnormality, strongly suggesting an acute process rather than chronic aneurysmal degeneration.

More recent reports highlight the concept of contained rupture and its variable clinical presentation. Ponte et al^8^ described a chronic, indolent course due to effective retroperitoneal containment, whereas our patient presented more acutely, likely reflecting impaired tamponade in the setting of profound coagulopathy. Kuwano et al^9^ reported rupture in the context of giant cell arteritis, demonstrating that inflammatory processes also may contribute to aortic wall vulnerability. In contrast, our case is more consistent with focal atherosclerotic or ulcerative pathology, supported by discontinuity of mural calcifications on imaging. Although the patient remained initially hemodynamically stable, consistent with partial containment, severe coagulopathy likely increased the risk of progression to free rupture.

The relationship between anticoagulation and acute aortic pathology remains incompletely understood. In this case, the imaging appearance favors an acute structural aortic lesion, such as focal atherosclerotic or ulcerative wall disruption, occurring in temporal association with profound warfarin-associated coagulopathy. However, a direct causal link cannot be established from this report. Rather than initiating rupture, severe coagulopathy likely impaired hemostasis and amplified hemorrhage after wall disruption had occurred.

From a management perspective, juxtarenal aortic pathology presents significant technical challenges. Open surgical repair requires suprarenal aortic clamping and is associated with substantial risks, whereas endovascular repair often requires specialized grafts that are not universally available.^10^ Nonoperative management of aortic rupture is associated with extremely high mortality, particularly in older patients with complex anatomy. Medical management therefore focused on rapid reversal of anticoagulation and strict hemodynamic control, although outcomes with no definitive repair remain poor.

This case is particularly unique because it demonstrates 3 uncommon features occurring simultaneously: 1) extreme warfarin-associated coagulopathy with an unmeasurable INR, 2) imaging-confirmed contained juxtarenal aortic perforation, and 3) absence of preexisting aneurysmal disease on recent imaging. To our knowledge, the coexistence of these findings has not been well described in contemporary literature.

Taken together, this case highlights that although warfarin-associated coagulopathy is unlikely to be the primary cause of aortic rupture, it may have exacerbated hemorrhage from a structurally vulnerable aortic lesion.

## Conclusions

This case highlights a rare presentation of contained juxtarenal aortic perforation in the setting of severe warfarin-associated coagulopathy. Although anticoagulation alone is unlikely to precipitate large-vessel rupture, this report suggests that profound coagulopathy may exacerbate hemorrhage from an underlying structural aortic lesion. The absence of prior aneurysmal disease on recent imaging supports the possibility of an acute process in a previously nondilated vessel, likely related to focal wall vulnerability.

Clinicians should maintain a high index of suspicion for atypical sources of bleeding in patients with severe coagulopathy presenting with abdominal or back pain. Early imaging, prompt reversal of anticoagulation, and strict hemodynamic control remain essential. Further studies are needed to better understand the relationship between anticoagulation and acute aortic pathology.

## Patient Perspective

Due to the patient's clinical condition and subsequent transition to hospice care, a direct patient perspective could not be obtained. The patient's family expressed appreciation for the care provided and the efforts of the clinical team.

### Ethical Approval

Ethical approval was not required for this study, as it describes a single patient case report without inclusion of identifiable patient information. Informed consent for publication was obtained.

### Consent

Written informed consent for publication of this case and accompanying images was obtained from the patient's authorized representative.

### Data Availability Statement

Data supporting the findings of this study are available from the corresponding author on reasonable request.

**Take-Home Messages**

• In patients with severe coagulopathy and unexplained abdominal or back pain, early cross-sectional imaging is essential to exclude life-threatening arterial pathology beyond typical bleeding sources.
• Initial management of suspected acute aortic injury should prioritize rapid anticoagulation reversal and strict blood pressure control, particularly when definitive intervention is limited by anatomy or patient factors.

## Funding Support and Author Disclosures

The authors have reported that they have no relationships relevant to the contents of this paper to disclose.
